# Assessment of Two Diabetes Point-of-care Analyzers Measuring Hemoglobin A1c in the Peruvian Amazon

**DOI:** 10.29024/aogh.2368

**Published:** 2018-11-05

**Authors:** Anthony T. Saxton, J. Jaime Miranda, Ernesto J. Ortiz, William Pan

**Affiliations:** 1 Duke Global Health Institute, Duke University, Durham, NC, US; 2 Miller School of Medicine, Miami University, Miami, FL, US; 3 CRONICAS Center of Excellence in Chronic Diseases, Universidad Peruana Cayetano Heredia, Lima, PE; 4 School of Medicine, Universidad Peruana Cayetano Heredia, Lima, PE; 5 Nicholas School of the Environment, Duke University, Durham, NC, US

## Abstract

**Background:**

With an estimated 174 million undiagnosed cases of diabetes mellitus worldwide and 80% of them occurring in low- and middle-income countries an effective point-of-care diagnostic tool is key to fighting this global epidemic. Glycated hemoglobin has become a reliable biomarker for the diagnosis and prognosis of diabetes.

**Objective:**

We assessed two point-of-care (POC) analyzers in multi-ethnic communities of the Amazon Rainforest in Peru where laboratory-based glycated hemoglobin (HbA1c) testing is not available.

**Methods:**

203 venous blood samples were tested for HbA1c by Afinion and DCA Vantage analyzers as well as a Premier Hb9210 high-performance liquid chromatography (HPLC) method as the reference standard. The coefficient of variation (CV) of each device was calculated to assess assay imprecision. Bland-Altman plots were used to assess bias. Ambient temperature, humidity, and barometric pressure were also evaluated for their effect on HbA1c results using multivariate regression.

**Findings:**

There was a wide range of HbA1c for participants based on the HPLC test: 4.4–9.0% (25–75 mmol/mol). The CV for the Afinion was 1.75%, and 4.01% for Vantage. The Afinion generated higher HbA1c results than the HPLC (mean difference = +0.56% [+6 mmol/mol]; *p* < 0.001), as did the DCA Vantage (mean difference = +0.32% [4 mmol/mol] *p* < 0.001). Temperature and humidity were not related to HbA1c; however, barometric pressure was associated with HPLC HbA1c results for the Afinion.

**Conclusions:**

Imprecision and bias were not low enough to recommend either POC analyzer for HbA1c determinations in this setting.

## Background

Diabetes mellitus is a global health crisis with an estimated 174 million people undiagnosed [1]. More than 80% of these unrecognized cases occur in low- and middle-income countries (LMICs) [2]. In addition to blood glucose measurements, one of the methods for diagnosing and managing diabetes is to analyze glycated hemoglobin (HbA1c) using laboratory-based instruments such as high-performance liquid chromatography (HPLC). HbA1c is an ideal biomarker for monitoring diabetes treatment as it indicates glycemic control from the previous three to four months and, unlike glucose tests, does not require pre-test fasting or measurements over two hours, has greater pre-analytical stability and less daily heterogeneity [3].

The use of HPLC to measure HbA1c in rural LMICs settings is largely untenable due to economic and geographic barriers. Cost of sample collection, transportation, and processing is high, locations with appropriate laboratory standards are not easily accessible, and protocols for storage and transport of samples to central labs face significant challenges [4]. Point-of-care (POC) analyzers have been developed that allow healthcare providers to obtain rapid HbA1c results on-site with significantly fewer logistical challenges as the HPLC method. POC results are often used to supplement clinical laboratory testing, but there is increasing interest to use them for screening as well as diagnostic and therapeutic monitoring [5]. In clinical laboratory settings, some POC analyzers have been shown to perform as well as laboratory-based methods [6]. However, questions remain as to how accurate they perform in different environments, particularly in LMIC settings where environmental conditions are less controllable than a clinical laboratory.

The Afinion™ AS100 Analyzer (Alere Technologies) and DCA Vantage™ Analyzer (Siemens Medical Solutions Diagnostics) are National Glycohemoglobin Standardization Program (NGSP) certified POC devices for evaluating HbA1c [7]. Both devices have shown low rates of bias and imprecision from standardized testing by the College of American Pathologists (CAP) [8]. Although healthcare providers in LMICs have recognized their potential for clinical use in low-income settings, very little is known about diabetes POC device performance in these environments where operating conditions vary significantly from controlled laboratory settings [9]. To our knowledge, no studies have investigated the performance of these HbA1c POC devices in multi-ethnic Amazonian groups or anywhere in South America. The increasing risk and continued undercount of diabetes in the Peruvian Amazon combined with economic and geographic barriers to HPLC diagnostics make this area ideal for research to determine the potential implementation of POC analyzers. The aim of this study was to evaluate the performance of the Afinion and DCA Vantage POC HbA1c analyzers for diabetes screening in an Amazonian region of Peru and compare the results to an NGSP-certified HPLC analyzer.

## Methods

### Setting

A cross-sectional study was performed in communities surrounding the Amarakaeri Communal Reserve in the southern Peruvian Amazon region of Madre de Dios (MDD). MDD is home to over 137,000 people, with several indigenous tribes disbursed throughout the area [10]. Medical care is provided primarily in government-run health posts of variable quality and resources.

**Figure 1:**
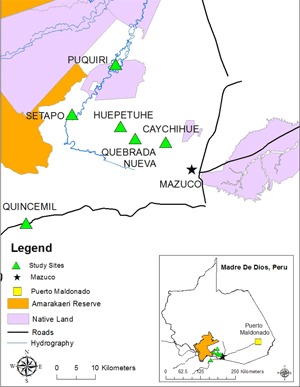
**Map of study sites and location where HbA1c values were obtained using point-of-care analyzers.** Green triangles represent the location of the six communities selected for this study, and the black star represents the site of HbA1c analysis using the point-of-care analyzers.

This study was embedded within the larger Amarakaeri Reserve cohort study, designed to evaluate the impact of natural resource extraction (natural gas and gold) on human and environmental health. The cohort study enrolled 1,122 households from 23 communities surrounding the reserve that are affected directly and indirectly by mining activities (Figure 1). This POC device ancillary study enrolled participants in six gold-mining communities in MDD: Huepetuhe, Quebrada Nueva, Caychihue, Setapo, Puquiri, and Quince Mil (Figure 1). These communities were selected based on: (1) their proximity to the city of Mazuco where all POC testing was performed; and (2) recommendations from the Regional Ministry of Health that these towns were believed to have the higher rates of diabetes than the reported 2.5% prevalence in the Peruvian jungle areas due to rapid urbanization and gold mining that has introduced a western diet to MDD [11].

### Point-of-care analyzers

The Afinion™ AS100 Analyzer (Alere Inc., Waltham, MA, USA) is a POC device that utilizes boronate affinity chromatography to measure the total percentage of glycation using a 1.5 μL sample of blood [12]. It has a reported HbA1c range of 4.0–15.0% and produces results in three minutes. Operating conditions are between 15–32°C (59–89°F) with 10–90% humidity for the analyzer, and 18–30°C (64–86°F) for the test cartridge [12,13].

The DCA Vantage™ Analyzer (Siemens Medical Solutions Diagnostics, Tarrytown, NY, USA) uses an immunoassay based on antibodies binding to glycated hemoglobin tetrapeptide or hexapeptide molecules using a 1.0 μL sample of blood [5]. It has a reported HbA1c range of 2.5%–14.0% and produces results in six minutes [14]. Operating conditions are between 15–40°C (61–104°F) with 15–90% relative humidity [14]. Both instruments utilize reagent cartridges that are used to collect samples of either capillary or venous blood and are inserted in the device for analysis.

### Sample design

Households with at least one woman of child-bearing age (15–49 years) were eligible for selection in this study. Community maps were obtained for Huepetuhe, Quebrada Nueva, and Quince Mil to draw a random sample. Since there were less than 75 households in Caychihue, Puquiri, and Setapo, each household with a woman of child-bearing age was approached to participate. Selected households that met the eligibility criterion were introduced to the study by trained fieldworkers, and family members were asked if they would like to participate. If consent was obtained, surveys were administered and biomarker samples were collected from the mother of the sentinel family unit, her spouse, and any children aged 12 and under. Venous blood samples were collected in K2 collection tubes containing edetic acid (EDTA) as a preservative. The tubes were labeled with the patient’s name, unique identifying number, and date, then placed in a Credo Cube™ cold box to store the samples between 2–8°C for up to ten days. Capillary blood samples were also drawn and anemia testing was performed with a HemoCue® POC device.

### Point-of-care testing

Batches of venous blood samples were delivered approximately twice per week to the town of Mazuco, where they were tested with the Afinion and DCA Vantage POC analyzers. Per manufacturer recommendations, before commencing tests on the participant samples each POC analyzer was initially verified to be in proper working condition by testing controls purchased from the manufacturers. Since HbA1c measurement can be affected by high temperature, the heat-sensitive color pads on the front of each box of DCA Vantage reagents was checked to ensure that the maximum temperature limit was not exceeded during transportation and storage. In addition to recording data on the HbA1c level of each sample, data was collected on the temperature, relative humidity, and barometric pressure of the room at the time of testing using the Ambient Weather WS-110 Wireless Weather Station. Furthermore, a single blood sample near the 6.5% (48 mmol/mol) diagnostic threshold for diabetes was measured 14 consecutive times using both POC devices.

### Laboratory testing

After obtaining HbA1c levels from the POC analyzers, the samples were stored at a 20°C cold chain and shipped to the Medlab Laboratory in Lima, Peru, where HbA1c levels were obtained using HPLC via the Premier Hb9210™ Analyzer (Trinity Biotech, Wicklow, Ireland). Control samples with HbA1c values of 5.5% and 11.5% were tested daily on the Premier Hb9210 to ensure proper calibration of the analyzer. All control testing measurements were within a 0.2% and 0.3% deviance for the 5.5% and 11.5% controls, respectively.

### Statistical methods

The analytical objectives were to measure imprecision of each POC device and to compare their HbA1c results to those obtained from the Premier Hb9210 analyzer. HbA1c was reported in Diabetes Control and Complications Trial (DCCT) aligned units (%) and converted to International Federation of Clinical Chemistry (IFCC) units (mmol HbA1c per mol unglycated hemoglobin) using the following master equation [15]:

{\rm{IFCC}} = ({\rm{NGSP}} - 2.15)/0.0915

Imprecision was evaluated from the sample of 14 repeated measurements using the coefficient of variation (CV), calculated as:

CV = s{\rm{/}}\bar x

where *s* is the sample standard deviation and \overline x is the mean of the duplicates. CVs were calculated with DCCT units using Microsoft® Excel 2011 (Microsoft Corp.). A 2% CV cutoff was used to categorize devices as meeting the generally accepted performance criterion for imprecision [6].

POC and HPLC results were compared by examining Pearson correlation coefficients from univariate regressions of paired samples and paired t-tests. Bland-Altman plots were used for bias assessments. Univariate regression was performed to test whether the bias was constant across the range of HbA1c concentrations.

Ambient temperature, humidity, and barometric pressure were evaluated for their effect on HbA1c results. A multivariate regression predicting HPLC’s HbA1c with results from each POC device and the ambient measurements was performed. Backwards selection procedures were used to remove non-predictive variables from the model. All statistical analyses were performed with STATA v13.1 (StataCorp LP).

### Ethical considerations

Approval for this study was obtained from the Institutional Review Board of the Universidad Peruana Cayetano Heredia. Children 12 and older provided assent, and all adult participants and parents of child participants provided informed consent and were advised on the research aims and objectives, rationale, expected benefits, and rights of participants.

## Results

HbA1c results using the Afinion and DCA Vantage POC analyzers and the Premier Hb9210 HPLC analyzer were obtained for 203 individuals. 137 participants were female (72%) with an average age of 36.6 years (range 12–75 years). Hemoglobin concentrations were obtained for 196 of the 203 participants, with 38 individuals (19.3%) having levels under 120 g/L for women and 130 g/L for men, indicating anemia. Anemia was more common in women (21.4%) than men (13.7%). Data about malaria was available for 198 participants, and 11 individuals (5.6%) reported being diagnosed with malaria in the past year.

The HbA1c values ranged from 4.4% (25 mmol/mol) to 9.0% (75 mmol/mol) per the HPLC analyzer (Table 1). Sixteen of the 203 samples tested by the Afinion reported an error code indicating that the blood sample had hemolyzed to a level that would interfere with analysis. No error codes were seen with the DCA Vantage or the HPLC method.

**Table 1:** HbA1c concentration (% DCCT units) by method.

	Afinion (n = 187)	DCA Vantage (n = 203)	HPLC (n = 203)

Mean (standard deviation)	5.8 (0.5)	5.5 (0.5)	5.2 (0.5)
Range	5.0–9.6	4.7–9.1	4.4–9.0

For the imprecision protocol, the mean HbA1c value from the 14 repeated measurements of a single sample was 6.69% (50 mmol/mol) for the Afinion, and 6.37% (46 mmol/mol) for the DCA Vantage. The sample standard deviation was 0.117% for the Afinion, and 0.255% for the DCA Vantage. The corresponding CV was lower for the Afinion (1.75%) than the DCA Vantage (4.01%).

**Figure 2:**
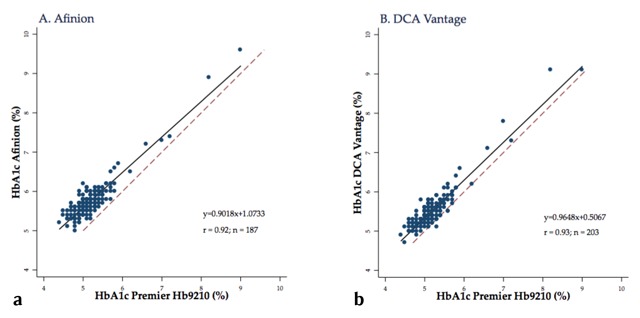
**Correlation between each point-of-care analyzer to the Premier Hb9210 for measuring HbA1c (DCCT units).** Blue dots represent individual samples, the dotted red line represents the line of identity x = y, and the solid black line is the regression line. Abbreviations: HbA1c, hemoglobin A1c; DCCT, Diabetes Control and Complications Trial.

The correlation of HbA1c values between the HPLC and POC analyzers were slightly higher for the DCA Vantage than Afinion. Correlation with the Afinion was 0.92, while correlation for the DCA Vantage was 0.93 (Figure 2a, b).

Bias relative to the HPLC test for the Afinion was +0.56% (+6 mmol/mol) (95% CI 0.53% to 0.59% [6 mmol/mol]), *p*-value < 0.001 by paired *t* test. The bias between the DCA Vantage and the HPLC analyzer was +0.32% (+4 mmol/mol) (95% CI 0.30% to 0.35% [3–4 mmol/mol]), *p*-value < 0.001. The bias observed was constant across the range of HbA1c concentrations for the DCA Vantage (*p* = 0.190), but not for the Afinion (*p* < 0.001), where bias increased as HbA1c levels decreased.

**Figure 3:**
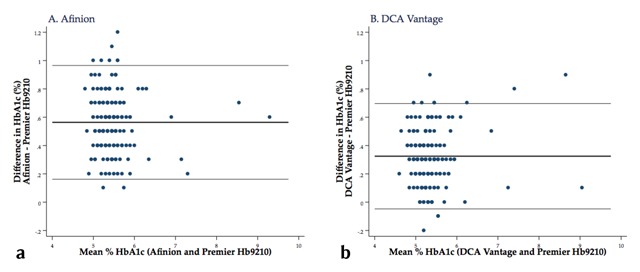
**Bland-Altman plot of the differences in HbA1c measurement (using DCCT units) between each point-of-care analyzer and the Premier Hb9210 by mean HbA1c level.** Blue dots represent individual samples. The horizontal black lines represent the bias (mean difference between the point-of-care analyzer and the Premier Hb9210) and its limits of agreement. Abbreviations: HbA1c, hemoglobin A1c; LOA, DCCT, Diabetes Control and Complications Trial.

The Bland-Altman limits of agreement (LOA) between the Afinion and the HPLC analyzer were 0.16% to 0.97% (2 to 11 mmol/mol), indicating that an individual from the studied population would have an expected difference within this range (Figure 3a). The LOA for the DCA Vantage and the HPLC analyzer was 0.05%–0.70% (1–8 mmol/mol) (Figure 3b).

Temperature ranged from 23.9–28.4°C (median 26.4°C), humidity ranged from 0.70–0.91% (median 0.83%), and barometric pressure ranged from 28.33–28.48 inHg (median 28.48 inHg). From the regression analysis, temperature and humidity were not related to HbA1c results for either POC device; however, barometric pressure was associated with the Afinion HbA1c. A one-unit increase in barometric pressure was associated with a 0.342% (DCCT units) increase in HbA1c (95% CI 0.050–0.634%, *p* = 0.022).

## Discussion

In this study we compared the bias of the Afinion and DCA Vantage POC analyzers with the Premier Hb9210 HPLC analyzer to measure HbA1c for diabetes screening in six Amazonian communities, and also measured the imprecision of the POC analyzers. Despite a high correlation observed between the POC analyzers and the laboratory-based HPLC method, a significant positive bias was detected for both POC analyzers. The range widths estimated for the LOAs for both devices were similar, but the DCA Vantage range included a mean difference of 0 while the Afinion did not. Both POC analyzers would have correctly categorized all five participants with diabetes using a 6.5% HbA1c threshold. However, amongst participants with an HPLC-confirmed HbA1c below 6.5%, the DCA Vantage incorrectly categorized one person as being in the diabetic range and the Afinion incorrectly categorized four people. In practice, this may lead to unnecessary medicalization and create unsustainable burdens for the healthcare system if the POC devices were used for HbA1c determination.

Peterson et al. [16] have also reported significant biases for the Afinion and the DCA Vantage, and Malkani et al. [17] also found a significant bias for the DCA Vantage. However, after programming the slope and intercept from their regression analyses into the DCA Vantage analyzer, they were able to reduce the average difference in subsequent testing to less than 0.2% HbA1c, and 0.0000% HbA1c, respectively. If POC testing is implemented in MDD, this could be a useful feature for the regional ministry of health to incorporate to harmonize the results between the DCA Vantage and laboratory-based instruments throughout the healthcare system.

The generally accepted performance criterion for imprecision is an intra-laboratory CV < 2% in DCCT units (<3% in SI units) [6]. We found that the CV for the Afinion (1.75% in DCCT units) met these standards, but the DCA Vantage (4.01%) did not. In practice, a low CV is important for clinicians to determine whether changes in HbA1c results over time reflect clinically significant changes in a patient’s glycemic status. A 0.5% (5 mmol/mol) difference in HbA1c is commonly used as an indication to adjust therapeutic options, although ultimately a statistically significant change in the health status of the patient should depend on the device’s CV and the within-person biological variation for HbA1c [6].

A review of the literature reveals that the CV we found for the Afinion is consistent with values reported in other studies, which range from 0.5% [16] to 3.1% [5], although those were all conducted in high-income countries including the Netherlands, United States of America, Norway, Spain, France, and South Korea. The CV that we found for the DCA Vantage is slightly higher than values previously published, which range from 1.55% [18] to 3.74% [19]. The direction and magnitude of the bias for these POC analyzers varies. The bias reported in the literature ranges from 1.1% [20] to 0.8% [16] (DCCT units) for Afinion and 0.53% [17] to 0.9% [16] for the DCA Vantage. Some studies have found that the Afinion produced lower HbA1c measurements than HPLC methods, [5,6,20,21,22,23] while others report higher HbA1c measurements [6,16,20,23,24,25,26]. Eight studies have demonstrated lower results from the DCA vantage compared to HPLC methods [5,6,17,18,23,25,27,28] and five have reported higher values [6,16,23,26,27]. To our knowledge, the only study to test the bias of the Afinion or DCA Vantage in an LMIC or tropical environment was performed by Wan Mohd Zin et al. in Malaysia [24].

For communities in the Peruvian Amazon, access to laboratory diagnostics for clinical care remains lacking. POC testing represents an opportunity for health posts to obtain clinical information that would otherwise be unavailable for patients due to financial and transportation constraints. Information to help diagnose and manage treatment for patients with diabetes will be particularly important in South and Central America, where it is projected that the number of people with the disease will increase 65% by 2040 [2]. Importantly, we found that nearly 1 in 5 participants in this study were anemic and 1 in 18 self-reported a previous case of malaria in the past year, which will directly affect hemoglobin concentrations and produce results that are unreliable for diagnostic determinations using any kind of HbA1c assay. If HbA1c testing is introduced in the Peruvian Amazon, we recommend the integration of routine proficiency testing amongst clinics to ensure awareness of other health conditions that may affect HbA1c results no matter how they were obtained, and also to improve consistency and enhance reliability of the results throughout the region.

While we were able to demonstrate the bias and imprecision of two POC analyzers compared to an NGSP-certified HPLC method, we acknowledge there were a few limitations with our study. First, interference from hemoglobin variants was not evaluated. The Afinion and DCA Vantage are unaffected by most common variants (HbS, HbC, HbE, HbD heterozygotes) but HbF levels greater than 10–15% can interfere with results [29]. Also, since all reagent cartridges were from a single lot for each analyzer, we were unable to test potential lot-to-lot variability, which can be an additional source of error in clinical use. Although we followed manufacturer recommendations in the storage of venous whole blood, the use of samples collected in-home and processed in a central location deviates from routine clinical practice and might introduce error to the analysis. In practice, a patient would normally travel to a clinic for a POC assay to be performed on-site and immediately after collection. Finally, there were not enough patients in this study around the 6.5% HbA1c diagnostic threshold to provide a definitive recommendation for or against the use of these POC devices for diagnostic purposes. Since the HbA1c results were from a largely non-diabetic sample, with only five of 203 participants having HbA1c levels indicative of a diabetes diagnosis, the overall results cannot be extrapolated to the diabetic population from this cohort.

In short, in settings such as hard-to-reach populations and indigenous groups where there is a high need to initiate diabetes prevention efforts, POC devices provide the appeal of simplicity of use and can potentially lower diabetes-related costs compared with laboratory-based methods. However, in this study we demonstrated that the DCA Vantage did not meet generally accepted performance criteria for HbA1c measurement precision, and both analyzers showed a large positive bias that may lead to unnecessary medicalization if utilized in this setting.
